# Efficacy of a needle-free blood collection device (PIVO Pro) for avoiding contaminated blood culture sets in Australian emergency departments: protocol for an adaptive group sequential randomised controlled trial

**DOI:** 10.1136/bmjopen-2026-125374

**Published:** 2026-09-28

**Authors:** Josephine Lovegrove, Andrea Valks, Daner Ball, Patrick N A Harris, Andrew Martin, Gaurav Langan, Sally Havers, Jonathan Javier Vico Da Silva, Gerben Keijzers, Barbara Read, Jason Chan, Karen Furlong, Jessica Schults, Claire Rickard

**Affiliations:** 1 NHMRC Centre of Research Excellence in Wiser Wound Care, Griffith University - Gold Coast Campus, Southport, Queensland, Australia; 2 School of Nursing, Midwifery and Social Work and Frazer Institute, The University of Queensland, Herston, Queensland, Australia; 3 Herston Infectious Diseases Institute, Metro North Hospital and Health Service, Herston, Queensland, Australia; 4 Frazer Institute, Faculty of Health, Medicine and Behavioural Sciences, The University of Queensland, Herston, Queensland, Australia; 5 Department of Microbiology, Pathology Queensland, Royal Brisbane and Women’s Hospital, Herston, Queensland, Australia; 6 ULTRA, The University of Queensland’s Clinical Trials Capability, Herston, Queensland, Australia; 7 Department of Emergency Medicine, Gold Coast Hospital and Health Service, Southport, Queensland, Australia; 8 Bond University Faculty of Health Sciences and Medicine, Gold Coast, Queensland, Australia; 9 School of Medicine and Dentistry, Griffith University - Gold Coast Campus, Southport, Queensland, Australia; 10 Health consumer representative, The University of Queensland, Brisbane, Queensland, Australia; 11 Emergency Department, Redcliffe Hospital, Redcliffe, Queensland, Australia; 12 The University of Queensland - St Lucia Campus, Brisbane, Queensland, Australia; 13 Caboolture Hospital, Caboolture, Queensland, Australia

**Keywords:** Clinical Trial, Emergency Departments, INFECTIOUS DISEASES, Adult pathology, Microbiology

## Abstract

**Introduction:**

Blood cultures are essential for diagnosing bloodstream infections and guiding antimicrobial therapy, but sample contamination can influence test sensitivity and treatment decision-making. The ‘PIVO Pro’ is a needle-free device which allows blood collection from a peripheral intravenous catheter (PIVC), with its use potentially avoiding sample contamination and decreasing the amount of venepuncture instances. However, existing evidence is limited and rigorous trial data are needed. Our primary objective is to compare the efficacy of the PIVO Pro at PIVC insertion for avoiding blood culture contamination to standard practice (typically specimen collection via venepuncture or a newly inserted PIVC).

**Methods and analysis:**

The PIVO Trial is a multicentre, adaptive group sequential randomised controlled trial. Across four Australian emergency departments, an estimated sample of 1148 adults (≥18 years) with a blood culture request, who also require a ≥22-gauge PIVC, will be included. Patients receiving intravenous antimicrobials prior to sample collection will be excluded. Randomisation will be 1:1 to PIVO Pro (one set of blood cultures drawn using PIVO Pro on PIVC insertion, subsequent sets drawn via venepuncture) or control (standard practice for all blood culture sets). The primary outcome is contamination (yes/no) in the target blood culture specimens (with all positive cultures independently assessed by blinded reviewers and classified as contaminated or ‘true positive’). Secondary outcomes include bottle underfill (assessed using blinded pathology processes), culture quality (composite of non-contamination and adequate fill) and rate of antibiotic use. The primary estimand compares contamination rates between arms using the PIVO-collected or first PIVC-drawn set from the initial collection episode (target sets), under a treatment-policy strategy, estimated using log-binomial regression. The design accommodates an interim analysis at 2/3 of planned total information. Recruitment commenced in August 2025.

**Ethics and dissemination:**

Ethics approval was obtained from Metro North Health Human Research Ethics Committee. Results will be disseminated via peer-reviewed publication and conference presentations.

**Trial registration number:**

NCT06806709, 4 January 2025, https://clinicaltrials.gov/study/NCT06806709

**Protocol version:**

V.1.5, August 2026.

**STRENGTHS AND LIMITATIONS OF THIS STUDY**

- Adults requiring blood cultures and a peripheral intravenous catheter will be included, with emergency department clinicians performing the procedures to reflect pragmatic, real-world practice.
- Central trial staff and ‘Master Trainers’ will closely support site-based research nurses, with the proximity of sites facilitating visits and coverage across health services if required.
- Research nurses will train clinicians on device use, perform recruitment and randomisation, collect relevant data and monitor protocol compliance and any adverse events.
- While the nature of the intervention prevents clinician and patient blinding, robust data collection from observations, pathology reports and clinical notes, with blinded primary outcome assessors and statisticians, will reduce performance and ascertainment bias.
- Primary estimand comparisons of blood culture target sets drawn using the PIVO Pro in each intervention patient, to those drawn via a peripheral intravenous catheter in each control patient, will detect intervention effects.

## Introduction

 Blood cultures are a diagnostic test used to detect microorganisms in the blood (ie, bloodstream infection), but collection is commonly compromised by two key challenges: sample contamination and inadequate bottle fill (underfill).^1^ Contamination refers to the introduction of skin or environmental bacteria into the sample during collection or processing, resulting in the specimen being labelled as ‘contaminated’ (and therefore unusable), or a ‘false-positive’ outcome which may trigger unnecessary antibiotic use, hospital stay and increased costs.^2^ Conversely, bottle underfill, with less blood volume in which microorganisms can be detected, results in reduced sensitivity which may produce a ‘false negative’ and delay appropriate treatment.^2^ Many patients requiring a blood culture also have a peripheral intravenous catheter (PIVC) inserted for therapy; however, it has long been recommended that PIVCs should not be used for blood sampling due to potential for haemolysis and contamination.^3^ This means patients receive multiple painful venepunctures for both PIVC insertion and blood sampling, which is compounded when compromised blood culture samples necessitate additional draws.

The emergence of specialised needle-free blood collection devices could enable the drawing of blood from PIVCs at any time without haemolysis, thus increasing patient comfort by reducing venepunctures.^4^ The PIVO Pro Needle-free Blood Collection Device (Becton, Dickinson and Company; Franklin Lakes) advances a flexible internal flow tube through the patient’s PIVC to access a fresh blood sample beyond the PIVC tip. There is supporting evidence that PIVO is comparable or superior to venepuncture^5–7^ and central line collection^8^ for yielding non-blood culture samples without or with an improved degree of haemolysis, clotting and preanalytical errors, while avoiding negative impacts on PIVC dwell time and replacement rates. However, there are limitations to previous research such as the use of non-randomised and retrospective study designs. Nonetheless, patients report a better experience with use of the PIVO compared with venepuncture^5^ and significantly less pain, resulting in a preference for its use.^6^

Another key result of one retrospective study^9^ in two hospitals who implemented PIVO for adult blood sampling from existing PIVCs was a significant decrease in blood culture ‘commensal events’ (isolation of an organism in the Centers for Disease Control (CDC) commensal list, which likely represents contamination) when comparing 1-year prior to and 1-year post PIVO implementation (hospital one absolute percentage decrease 26%; hospital two absolute percentage decrease 46%).^9^ In comparison, 71 ‘control’ hospitals who had not implemented PIVO saw an absolute percentage decrease of only 12% across the same time period. This suggests that use of the PIVO Pro may decrease blood culture contamination. However, while the PIVO Pro is used routinely (standard care) in some US hospitals, rigorous trial data are needed to inform broader uptake. In Australia, many state and territory health department policies only permit PIVC blood sampling via a newly inserted device.^10^ Theoretically, for newly inserted PIVCs, the PIVO Pro would bypass the internal PIVC which may have been contaminated on insertion through the skin, to collect a clean blood culture sample from beyond the tip, while also avoiding complications which may be associated with PIVC blood draw. Therefore, our primary aim is to examine whether use of the PIVO Pro at PIVC insertion, compared with standard practice, avoids blood culture contamination.

## Methods and analysis

### Objectives

The primary objective is to compare the efficacy of the PIVO Pro at PIVC insertion to avoid contaminated blood culture sets, to standard practice for blood culture set draw.

The secondary objectives are to compare the efficacy of the PIVO Pro at PIVC insertion, in comparison to standard practice to achieve adequate fill volume of blood culture bottles; to improve the overall quality of blood culture set samples (composite of: non-contamination and adequate fill volume); and reduce antibiotic use.

We hypothesise that use of the PIVO Pro at PIVC insertion will be superior in avoiding contaminated blood culture specimens when compared with standard practice.

### Study design and setting

The PIVO Trial is a multicentre, adaptive group sequential RCT with an interim analysis at two-thirds of planned recruitment to inform potential modifications to trial design. Reporting of this protocol aligns with the Standard Protocol Items: Recommendations for Interventional Trials (SPIRIT) Statement^11^ (online supplementary 1).

The trial will be conducted in four emergency departments (where many PIVCs are newly inserted and blood cultures drawn and blood samples are commonly collected from newly inserted PIVCs^12^) across two health services in South-East Queensland, Australia (Gold Coast Health: Gold Coast University Hospital, Robina Hospital; Metro North Health: Caboolture Hospital, Redcliffe Hospital). Trial definitions are presented in table 1. Recruitment commenced in August 2025.

**Table 1:** The PIVO Trial definitions

Term	Definition
Blood culture set	One aerobic and one anaerobic blood culture bottle collected from a single blood draw.^1^
Blood culture episode	All blood culture sets collected at one point in time. For adults, at least two sets of BCs should be collected from a different bodily location, to increase the chance of having at least one non-contaminated specimen.^28^ Additional (3+) sets may be collected in some circumstances, such as where a central line is in situ.^29^ Only one set may be taken in some clinical circumstances. All of these are consistent with current policy and practice at the study sites.
Blood culture bottle underfill	Underfilling of blood culture (< the optimal fill volume which is 10 mL; BACT/ALERT adult bottles used by study sites in standard care) in at least one bottle of a set. Use of paediatric bottles (typically due to poor blood flow or difficult intravenous access) will be considered a protocol deviation.
Positive blood culture	Detection of the presence of bacteria or fungi in the blood by culture in ≥1 blood specimens (whether from sample contamination or a true positive result).
Blood culture contamination	Laboratory identification of a common skin commensal in a single BC set in a BC collection episode,^19–23^ without identification of the same common skin commensal by culture in ≥2 blood specimens collected on separate occasions (assigned separate specimen numbers, processed individually and reported separately in the final laboratory report^23 24^), which would warrant reclassification to ‘true positive’ (see Outcome measures). Where unclear in laboratory results, the following criteria will be considered in the patient’s clinical context and applied to identify contamination: documentation in the medical record/discharge summary as ‘contaminated’, OR (2) a positive blood culture report that is not treated and patient signs and symptoms of infection resolving within 24 hours.
‘True positive’ blood culture	Detection of the presence of bacteria in the blood which is not categorised as contamination (eg, presence of a recognised bacterial or fungal pathogen which is not a common skin commensal by culture in ≥1 blood specimens; or presence of the same common skin commensal by culture in ≥2 blood specimens collected on separate occasions^23^; or categorisation of a ‘true positive’ based on assessment of clinical context, clinical documentation and/or untreated positive blood culture with symptoms resolving within 24 hours**.**

.BC, blood culture; PIVC, peripheral intravenous catheter.

### Study participants

Adults (≥18 years) who require blood cultures and insertion of a ≥22-gauge PIVC will be eligible. Patients who have already commenced intravenous antimicrobial medications or who have previously been enrolled will be ineligible.

### Sample size calculation

A sample size of 1148 participants will be randomised in a 1:1 ratio to achieve 90% power at the two-sided alpha of 5% assuming the failure probability (contamination of the blood culture set) is 4% in the control arm and 1% in the PIVO Pro arm. Failure probability in the control arm was estimated based on blood culture contamination rates reported in Australian emergency departments (range 2.1–5.5%, average 3.7%; blood culture set sample size range 3700–6953),^1 13–15^ and the observed rates reported by one study site (contamination ≈4%) for this calculation. PIVO Pro failure probability was estimated and considered realistic based on a previous study implementing the PIVO Pro (commensal percentage decrease 26% and 46% in two hospitals pre/post PIVO implementation; total commensal events, n=747; sample size not reported),^9^ a study examining use of a different but similar device which diverts the first potentially-contaminated blood away from the sample (contamination 0.22% with device, 1.78% without; 904 blood cultures per arm),^16^ and an unpublished laboratory simulation of sample contamination using the PIVO device (0%; unpublished, held by Becton, Dickinson and Company). Based on previous rates of blood culture requests in the study sites prior to trial planning, it is anticipated the sample size is achievable, and that recruitment would take approximately 12 months. Recruitment rates will be monitored by central research staff and a monthly target set. Sites will be given regular updates on their progress with benchmarking to other sites. If necessary and where identified, the investigators will discuss slower than anticipated recruitment with sites to identify solutions.

### Study procedures

Site-based research nurses will coordinate and perform study procedures locally, supported by a central trial project manager and ‘Master Trainers’. Master Trainers (n=2; each focused on one predominant health service) received prior extensive training in use of the PIVO Pro from the manufacturer (Becton, Dickinson and Company). The proximity of sites will facilitate travel and visits by central staff and trainers across health services if required for support or site coverage.

Master Trainers will be responsible for training the site research nurses in use of the PIVO Pro and for supporting site research nurses in study procedures and training site clinicians. The research nurses will be responsible for clinician training in PIVO Pro use (pre-trial in services, ongoing rapid bedside training to support sustained expansion in skilled clinicians across the trial to maintain recruitment capacity; with staff competency assessed using the PIVO Pro, and recorded in a training log), patient identification, recruitment, randomisation and data collection. PIVC insertion and blood culture draw (with/without PIVO) will be performed by the bedside clinicians per standard care.

#### Screening, randomisation and allocation concealment

Research nurses will screen patients presenting to the emergency department against the eligibility criteria. Once patient eligibility is confirmed, informed verbal consent using a predetermined script will be requested from the patient or their substitute decision-maker (where necessary and present). Patients will only be included and randomised if consent is obtained.

The research nurses will obtain the randomisation allocation for each patient immediately after study entry. Randomisation will be generated 1:1 to a study arm (table 2) at the bedside/treatment space by a computer-generated web-based central service (Research Electronic Data Capture; REDCap^17 18^), ensuring allocation concealment and the provision of urgent treatment. Randomisation will be stratified by hospital site. Following randomisation, PIVC insertion and/or blood culture draw and baseline data collection will commence.

**Table 2:** Study arms

Arm	Intervention
PIVO Pro arm	PIVC inserted per standard practice; target set collected via PIVO Pro; any additional sets collected by venepuncture from a different site (fall-back if the target set is missing: first drawn using venepuncture, peripheral intravenous catheter without PIVO Pro or other vascular access device).
Control arm	Collection of all BC sets using standard practice (typically one set each for venepuncture and one draw from newly inserted PIVC); target set is first PIVC-drawn set (newly inserted or existing; fall-back if the target set is missing: first drawn using venepuncture or other vascular access device).

BC, blood culture; PIVC, peripheral intravenous catheter.

#### Intervention

In both arms (table 2), PIVC insertion and blood culture draw will be undertaken by emergency department clinicians/the treating team who are already skilled in these tasks as part of standard practice, with the support of the research nurses.

In the control arm, all blood culture sets will be collected and processed using standard practice (typically one each for venepuncture/draw from a newly inserted PIVC). For newly inserted PIVCs, the devices used in standard practice in the study sites are BD Insyte Autoguard intravenous Cannula (with/without Blood Control Technology) 20GA (1.1×30 mm length) and 22GA (0.9×25 mm length), secured with 3M Tegaderm intravenous transparent film with border dressings. Blood specimens may be collected directly from the PIVC using a luer-lock syringe (BD Plastipak) or via a connector (BD Smartsite Needle-Free Valve) attached after insertion, with a needle attached to the syringe after draw to inoculate the blood culture bottles. Transfer devices are not typically used in place of a syringe.

In the PIVO Pro arm, one blood culture set will be collected using the PIVO Pro device from a newly inserted PIVC. The PIVO Pro will be used with the BD ExT Stabilised Extension Set with NearPort intravenous Access (allows connection of the PIVO Pro to any PIVC) and the ICU Medical Saf-T Holder (allows transfer of blood from the PIVO Pro into the blood culture bottles). PIVCs and dressings will be as per standard practice. The full procedure as a clinical skill assessment checklist is presented in online supplementary 2. Any further blood culture sets will be collected from a different body location using venepuncture, usually done per standard practice with the BD Vacutainer UltraTouch Push Button Blood Collection Set 23 or 21 G (0.6–0.8×19 mm×178 mm).

Blinding of patients and clinical staff is impracticable due to the visibility of the intervention; however, outcome assessors, laboratory staff and statisticians will be blinded with the primary endpoint additionally blind verified. Laboratory processes including reporting of results to clinicians will follow standard practice, with staff unaware that the specimen is being included in a trial. Data will be masked for blinded statisticians, while an independent unblinded statistician will prepare unmasked data for interim analysis.

#### Trial monitoring

Central research staff will remotely monitor recruitment, data entry and protocol compliance on an ongoing basis, with monthly monitoring site visits to source verify consent and data entry by the project manager. Monitoring reports will be provided to the trial investigators and Trial Steering Committee (monthly).

### Study outcome measures

#### Primary outcome measures

Blood culture contamination (defined as yes/no—measured as laboratory identification of a common skin commensal in a single blood culture set (see table 1) in an episode.^19–23^ As per CDC/National Healthcare Safety Network,^23^ should the same common skin commensal be identified by culture in ≥2 blood specimens collected on separate occasions (same or consecutive days; eg, on the same day as the trial blood culture set/s draw but several hours later) and the blood cultures are assigned separate specimen numbers, processed individually and are reported separately in the final laboratory report, the result will be reclassified as being a true positive^23 24^ (ie, not contaminated).

Should contamination of a blood culture set be unclear based on laboratory results and the above classification requirements, the following criteria will be considered in the patient’s clinical context and applied to identify contamination: (1) documentation in the medical record/discharge summary as ‘contaminated’, OR (2) a positive blood culture report that is not treated and patient signs and symptoms of infection resolving within 24 hours.

#### Primary outcome assessment

Two independent, blinded, expert outcome assessors will be provided with de-identified data for each participant with a positive blood culture to identify occurrence of the primary outcome (contaminated or a true positive). Disagreements between assessors will be resolved via subsequent discussion, or if necessary, arbitration by a third assessor. Outcome assessors will be clinical experts in infectious diseases or infection prevention.

#### Secondary outcome measures

The secondary outcomes are:

- Blood culture underfill—inoculation of blood into a blood culture bottle <10 mL (optimal fill volume for BACT/ALERT bottles used across sites as standard care) as reported by pathology reports (laboratory staff blinded).
- Blood culture set quality—measured as a composite of blood culture non-contamination and adequate fill volume.
- Antibiotic use (data captured by unblinded research nurses, but easily identifiable and verifiable in the medical records)—antibiotic use following blood culture draw (binary yes/no), antibiotic use change or cessation following blood culture results (yes/no) and reason for cessation, further/new treatment started (yes/no), reason for commencement of antibiotic.

### Data collection

The research nurses will perform baseline data collection on recruitment and during observation of blood culture collection by clinical staff. Baseline data will be captured from patient bedside observation, the medical record and clinician report, including patient demographics, comorbidities and medical history.

Participants will not be directly followed-up. However, follow-up data will be collected from the participant’s medical records following hospital discharge. This data will be captured from laboratory reports, medical/clinical documentation (all sites using electronic medical records) and the discharge summary.

All baseline and follow-up data (table 3) will be entered into standardised data collection forms hosted by REDCap, hosted by The University of Queensland.

**Table 3:** Data points

Baseline data (from patient bedside, medical records, clinician report)	Follow-up data (from laboratory reports, medical/clinical notes, discharge summary)
Trial identification Site. Eligibility and randomisation. Patient/clinical characteristics (from medical records, clinician report) Gender, age, skin type, body mass index, smoking history, employment status. Vital signs. Comorbidities. Triage category. Paralysis/central nervous system diagnosis. Current known infection. Suspected sepsis and source/s. Additional culture specimens collected. Recent antibiotic use. BC indication signs/symptoms. Difficult intravenous access status. PIVC and BC (from research nurse observation) Number of BC sets taken. For each BC set: Date/time. Vein blood drawn from. Drawer profession. Draw modality. Minimum fill volume met (anaerobic and aerobic bottle). Where BC set taken from newly inserted PIVC: Inserter profession (if different from BC drawer). PIVC gauge. PIVC and BC (from medical record, clinician report) Where BC set taken from pre-existing PIVC: Location of insertion (eg, ambulance, ED). Length of dwell in hours. Reason for removal. Previous use. Adverse events of special interest. Serious adverse events. Protocol deviations.	BC results BC results by set. Positive/negative. Organism identified. Hours until first positive. Modality of draw (PIVO, standard care). Underfill per laboratory results. Documentation of contamination in clinical notes/discharge summary. Treatment post BC set result. Time to symptoms resolving. Clinical characteristics Length of ED stay. Admission to hospital and length of stay. Nature of any treatment and treatment changes during care. Actual time to symptoms resolving. Subsequent BCs and other culture specimens during admission taken, date/time, positive/negative result, organism(s) if positive. Treatment (from medical record, clinician report) Commencement and nature of antibiotics/treatment post-BC including timing and duration. Other treatments. Adverse events of special interest. Serious adverse events. Protocol deviations.

BC, blood culture; ED, emergency department; PIVC, peripheral intravenous catheter.

### Safety considerations

Provision of care, including PIVC insertion and maintenance, will align with standard care and local policies and procedures. All blood culture results will be available as per usual practice to the treating clinicians. Any adverse events associated with a PIVC will follow standard treatment, reporting mechanisms and policy at each trial site. The PIVO Pro is approved for use in the USA by the Food and Drug Administration, and a Clinical Trial Notification was submitted to the Australian Therapeutic Goods Administration for use of the PIVO Pro and ExT Stabilised Extension Set with NearPort intravenous Access in this trial. It is therefore anticipated that the risk of adverse events associated with the device is minimal. Risk of user error, such as incorrect attachment of the PIVO Pro or incorrect blood draw, will be minimised by clinician training. The PIVO compatible connector will remain in situ following blood sampling for clinical use. While its use follows that of the connectors used in standard practice, education material will be provided to medical imaging and inpatient unit nurses. Any adverse events resulting from PIVO Pro attachment and use will be recorded, and any serious adverse events reported to the approving Human Research Ethics Committee (HREC), within the mandatory reporting time frame. There is no direct patient follow-up following blood culture collection; treatment and PIVC management will be continued as per standard practice. Thus, it is not anticipated that trial-related post-participation and ancillary care will be required.

An independent Data and Safety Monitoring Board (DSMB) will monitor accumulated unblinded safety and operational data and make recommendations to the Trial Steering Committee about trial continuation and modifications (see Statistical considerations: Interim analysis). The DSMB will comprise statistical, emergency medicine and infectious diseases experts (n=3).

### Statistical considerations

A detailed Statistical Analysis Plan will be finalised prior to database lock and unblinding and will provide further details of all planned analyses.

#### Interim analysis

The design allows for interim analysis at two-thirds (n=765) of the planned recruitment, which will be evaluated by the DSMB who will advise whether modifications to the design are warranted. The boundaries for rejecting the null hypothesis will be based on the Lan-DeMets O’Brien-Fleming approximation spending function (early stopping for efficacy). If the efficacy boundary is not crossed, conditional power will be calculated—with a value below 20% prompting consideration of a futility conclusion—and a blinded assessment of sample size assumptions conducted to inform any recommendations to modify the trial design. If evaluation of accumulated data suggests unreasonable harm in either arm at any review, a protocol amendment or arm suspension or termination may be recommended as appropriate. If none of these conditions are met, the board will recommend continuation.

The alpha spending function will guide how much of the total alpha is used for the interim analysis of the primary endpoint and the final analysis should the trial continue to that point. All secondary outcomes will be tested at the two-sided 5% alpha level with results being interpreted with due consideration, but without strong control of multiplicity.

#### Primary outcome

The primary analysis will estimate the treatment effect for the primary estimand in the intention-to-treat population, comprising all randomised participants. The outcome is a binary indicator of contamination in the target blood culture set as confirmed by the blinded verification committee. Intercurrent events, including use of an alternative collection method, will be handled using a treatment-policy strategy; if PIVO Pro was not used in the intervention arm or draw via a PIVC was not used in the control arm (target sets), set one (the lowest drawn) via venepuncture, PIVC or other vascular access device in the intervention arm and venepuncture or other vascular access device in the control arm, will be used as the ‘fall back’ target blood culture set. A separate per-protocol estimand will be evaluated in participants who received the allocated collection method, excluding those with such deviations.

Primary analysis will use a log-binomial regression model to estimate the relative risk between groups for the primary binary outcome, accounting for stratification. If non-convergence occurs, Poisson regression with robust variance will be used. Missing data will be handled using complete case analysis, with multiple imputation using chained equations conducted as a supplementary analysis if missingness exceeds 5%.

#### Secondary outcomes

Secondary outcomes will be analysed using the same modelling framework as the primary analysis where appropriate, with alternative methods used for non-binary outcomes according to their distribution and scale.

### Patient and public involvement

A patient consumer representative is an active trial investigator (BR), joining during trial planning and study document finalisation. The consumer provided review and feedback on study documents, including recruitment procedures and the verbal consent script. The consumer investigator is an ongoing member of the Trial Steering Committee monitoring recruitment and progress and will contribute to the interpretation of results to reflect on consumer priorities and experience, as well as dissemination. At results dissemination, consumer involvement throughout the trial will be reported using the Guidance for Reporting Involvement of Patients and the Public Checklist.^25^

## Ethics and dissemination

### Research ethics approval

Full ethical approval has been granted by the Metro North Health HREC (HREC/2024/MNHA/11299) and ratified by relevant university HRECs (University of Queensland 2024/HE002583; Griffith University 2025/010). The study will be conducted in accordance with the Declaration of Helsinki and Good Clinical Practice. The trial has been registered with ClinicalTrials.gov. The trial sponsor is The University of Queensland.

### Intercurrent events (protocol violations, deviations) and amendments

A violation of the protocol will be defined as a participant receiving the incorrect care based on trial arm. Violations will be recorded in the participant data collection forms and will be accounted for in the primary estimand.

A deviation of the protocol will be defined as a departure from the approved study protocol, document or process. A protocol deviation will also be recorded in the participant data collection forms and will be reported by sites to the principal investigator for assessment of significance. Deviations with the potential to impact on the integrity of the study, ethical acceptability or patient safety will be escalated to the HREC in a timely manner. Where deviations identify protocol issues requiring review, the protocol will be amended and submitted for approval to the HREC.

Any substantial change (changes likely to have significant impact on protection of individuals, research validity, results, quality and safety of devices and interpretation of documents) to the protocol will be submitted to the HREC for approval prior to implementation. Non-substantial changes (no significant impact on any trial aspects) will be communicated to the HREC in due course.

### Consent

Given the need for rapid assessment and treatment, including the draw of blood cultures, in the often very unwell trial population, verbal consent will be sought from potential participants (or their substitute decision-maker where necessary and present) following a predetermined script (online supplementary 3), prior to inclusion in the study and randomisation. Verbal consent in this trial aligns with the requirements of the Australian National Statement on Ethical Conduct in Human Research.^26^

### Confidentiality

To maintain patient confidentiality, all identifiable patient data (patient identifier in site enrolment log) will be held securely on-site, and no identifiable data will be given to the coordinating centre, analysts or investigators not at the study site. All data entered into REDCap will be tracked through a Study ID, only re-identifiable via the site enrolment log. All data viewed and exported out of REDCap will not be identifiable.

### Dissemination

Results will be disseminated through peer-reviewed open-access publication (following the Adaptive designs Consolidated Standards of Reporting Trial (CONSORT) Extension statement^27^) and conference presentations.

### Data sharing

Data (including the data dictionary), statistical coding and other materials (data collection forms, data management procedures, DSMB charter) are available from the authors on reasonable request and within the scope of trial ethical approval once the primary results have been published.

## Supplementary material

10.1136/bmjopen-2026-125374 online supplemental file 1
