# Supplementary material for: Efficacy of a needle-free blood collection device (PIVO Pro) for avoiding contaminated blood culture sets in Australian emergency departments: protocol for an adaptive group sequential randomised controlled trial
Source: BMJ Open. 2026 Sep 28;16(9):e125374. doi: 10.1136/bmjopen-2026-125374 (PMC13663724; doi:10.1136/bmjopen-2026-125374)
Supplement: Supplementary file 1 [file bmjopen-2026-125374.reviewer_comments.pdf]

PEER REVIEW HISTORY

BMJ Open publishes all reviews undertaken for accepted manuscripts. Reviewers are asked to complete a checklist review form and are provided with free text boxes to elaborate on their assessment. These free text comments are reproduced below.

ARTICLE DETAILS

Title (Provisional)

Efficacy of a needle-free blood collection device (PIVO Pro) for avoiding contaminated blood culture sets in Australian emergency departments: Protocol for an adaptive group sequential randomised controlled trial.

Authors

Lovegrove, Josephine; Valks, Andrea; Ball, Daner; Harris, Patrick N. A.; Martin, Andrew; Langan, Gaurav; Havers, Sally; Vico Da Silva, Jonathan Javier; Keijzers, Gerben; Read, Barbara; Chan, Jason; Furlong, Karen; Schults, Jessica; Rickard, Claire

---

VERSION 1 - REVIEW

**Reviewer**	**1**
**Name**	**Badrick, Tony**
**Affiliation**	**Royal College of Pathologists of Australasia, QAP**
**Date**	**07-Aug-2026**
**COI**	**None**

This protocol for a randomised controlled trial has a well-defined research question with a clearly described study design, setting, and procedures. The outcomes are clear and measurable with an independent process for blinding and statistical assessment, and the protocol follows the SPIRIT guidelines. The trial is of high interest clinically because of the issue of BC contamination. Overall, the protocol meets all requirements.

Some comments:

1. Is the restriction of the study centres being in one health network, with the same research nurses available a further advantage of the study?

2. What is the risk of not being able to access and train all relevant clinical staff?

3. It may be useful to provide the sample sizes of the studies used to determine the sample size on page 10.

4. Is the assumed failure probability 1% in the PIVO Pro arm optimistic?

5. How long is it estimated that it will take to reach the 1148 samples?

Reviewer 2

Name McFadden, Benjamin R.

Affiliation The University of Western Australia Faculty of Engineering Computing and Mathematics

Date 24-Aug-2026

COI None

Thank you for the opportunity to review this important protocol. The protocol is very well written and in my opinion, meets the standard for publication. I have no comments to add.

VERSION 1 - AUTHOR RESPONSE

Reviewer 1		
1. Is the restriction of the study centres being in one health network, with the same research nurses available a further advantage of the study?	1. Study centres were across two local health services, we have now clarified this in the manuscript.<br><br>2. However, their close location means that central research staff and trainers could support site-based research nurses across health services if needed, and we agree this was an advantage. We have now added this to the strengths and limitations and manuscript.	1. Study design and setting<br>2. Strengths and limitations of this study; Study procedures
2. What is the risk of not being able to access and train all relevant clinical staff?	The training approach was developed to minimise the risk of few staff being trained/competent and thus slow recruitment. Training can be done rapidly at the bedside as the trial progresses by site-based staff. This is reported in the second paragraph of the Study procedures section. We have also added to this section to state "to support sustained expansion in skilled clinicians	Study procedures

	across the trial to maintain recruitment capacity”.	
3. It may be useful to provide the sample sizes of the studies used to determine the sample size on page 10.	Sample sizes (where and as reported) have been added.	Sample size calculation
4. Is the assumed failure probability 1% in the PIVO Pro arm optimistic?	We consider this estimation realistic based on the reported previous research which informed it. We have added a note to state this is realistic in the relevant section.	Sample size calculation
5. How long is it estimated that it will take to reach the 1148 samples?	It was estimated during planning that recruitment would take approximately 12 months. This has been added to the manuscript.	Sample size calculation
Thank you for the opportunity to review this important protocol. The protocol is very well written and in my opinion, meets the standard for publication. I have no comments to add.	Thank you for your positive review, it is very much appreciated.	Not applicable
