# Supplementary material for: Efficacy of a needle-free blood collection device (PIVO Pro) for avoiding contaminated blood culture sets in Australian emergency departments: protocol for an adaptive group sequential randomised controlled trial
Source: BMJ Open. 2026 Sep 28;16(9):e125374. doi: 10.1136/bmjopen-2026-125374 (PMC13663724; doi:10.1136/bmjopen-2026-125374)
Supplement: Supplementary file 2 [file bmjopen-16-9-s001.docx]

**Supplementary 1. SPIRIT 2025 checklist of items to address in a randomized trial protocol***

| **Section / Topic** | **No** | **SPIRIT 2025 checklist item description** | **Reported on page no.** |
| --- | --- | --- | --- |
| **Administrative information** | | |  |
| Title and structured summary | 1a | Title stating the trial design, population, and interventions, with identification as a protocol | 1 |
|  | 1b | Structured summary of trial design and methods, including items from the World Health Organization Trial Registration Data Set | 1, 2 |
| Protocol version | 2 | Version date and identifier | 2 |
| Roles and responsibilities | 3a | Names, affiliations, and roles of protocol contributors | ScholarOne system, 25 End Matter Author contributions |
|  | 3b | Name and contact information for the trial sponsor | 18, 25 End matter |
|  | 3c | Role of trial sponsor and funders in design, conduct, analysis, and reporting of trial; including any authority over these activities | 18, 25-26 End Matter Funding statement |
|  | 3d | Composition, roles, and responsibilities of the coordinating site, steering committee, endpoint adjudication committee, data management team, and other individuals or groups overseeing the trial, if applicable | 6, 8, 9, 11-13, 16, 18 |
| **Open science** | | |  |
| Trial registration | 4 | Name of trial registry, identifying number (with URL), and date of registration. If not yet registered, name of intended registry | 2 |
| Protocol and statistical analysis plan | 5 | Where the trial protocol and statistical analysis plan can be accessed | Trial registration: 2,18  Protocol: submitted manuscript  SAP: 16 (notes to be finalised prior to database lock), data sharing 20 |
| Data sharing | 6 | Where and how the individual de-identified participant data (including data dictionary), statistical code, and any other materials will be accessible | 20 |
| Funding and conflicts of interest | 7a | Sources of funding and other support (e.g., supply of drugs) | 26 End Matter |
|  | 7b | Financial and other conflicts of interest for principal investigators and steering committee members | 26 End Matter |
| Dissemination policy | 8 | Plans to communicate trial results to participants, healthcare professionals, the public, and other relevant groups (e.g., reporting in trial registry, plain language summary, publication) | 2, 18, 20 |
| **Introduction** | | |  |
| Background and rationale | 9a | Scientific background and rationale, including summary of relevant studies (published and unpublished) examining benefits and harms for each intervention | 4, 5 |
|  | 9b | Explanation for choice of comparator | 4, 5, 6, 9, 10, 11 |
| Objectives | 10 | Specific objectives related to benefits and harms | 5, 6 |
| **Methods: Patient and public involvement, trial design** | | |  |
| Patient and public involvement | 11 | Details of, or plans for, patient or public involvement in the design, conduct, and reporting of the trial | 18 |
| Trial design | 12 | Description of trial design including type of trial (e.g., parallel group, crossover), allocation ratio, and framework (e.g., superiority, equivalence, non-inferiority, exploratory) | 6 |
| **Methods: Participants, interventions, and outcomes** | | |  |
| Trial setting | 13 | Settings (e.g., community, hospital) and locations (e.g., countries, sites) where the trial will be conducted | 6 |
| Eligibility criteria | 14a | Eligibility criteria for participants | 7, 8 |
|  | 14b | If applicable, eligibility criteria for sites and for individuals who will deliver the interventions (e.g., surgeons, physiotherapists) | n/a |
| Intervention and comparator | 15a | Intervention and comparator with sufficient details to allow replication including how, when, and by whom they will be administered. If relevant, where additional materials describing the intervention and comparator (e.g., intervention manual) can be accessed | 8-11 |
|  | 15b | Criteria for discontinuing or modifying allocated intervention/comparator for a trial participant (e.g., drug dose change in response to harms, participant request, or improving/worsening disease) | 15-17 |
|  | 15c | Strategies to improve adherence to intervention/comparator protocols, if applicable, and any procedures for monitoring adherence (e.g., drug tablet return, sessions attended) | 9-11 |
|  | 15d | Concomitant care that is permitted or prohibited during the trial | n/a |
| Outcomes | 16 | Primary and secondary outcomes, including the specific measurement variable (e.g., systolic blood pressure), analysis metric (e.g., change from baseline, final value, time to event), method of aggregation (e.g., median, proportion), and time point for each outcome | 11-15 |
| Harms | 17 | How harms are defined and will be assessed (e.g., systematically, non-systematically) | 14-16 |
| Participant timeline | 18 | Time schedule of enrollment, interventions (including any run-ins and washouts), assessments, and visits for participants. A schematic diagram is highly recommended (see Figure) | 13 |
| Sample size | 19 | How sample size was determined, including all assumptions supporting the sample size calculation | 8 |
| Recruitment | 20 | Strategies for achieving adequate participant enrollment to reach target sample size | 8 |
| **Methods: Assignment of interventions** | | |  |
| Randomization: |  |  |  |
| Sequence generation | 21a | Who will generate the random allocation sequence and the method used | 9 |
|  | 21b | Type of randomization (simple or restricted) and details of any factors for stratification. To reduce predictability of a random sequence, other details of any planned restriction (e.g., blocking) should be provided in a separate document that is unavailable to those who enroll participants or assign interventions | 9 |
| Allocation concealment  mechanism | 22 | Mechanism used to implement the random allocation sequence (e.g., central computer/telephone; sequentially numbered, opaque, sealed containers), describing any steps to conceal the sequence until interventions are assigned | 9 |
| Implementation | 23 | Whether the personnel who will enroll and those who will assign participants to the interventions will have access to the random allocation sequence | 9 |
| Blinding | 24a | Who will be blinded after assignment to interventions (e.g., participants, care providers, outcome assessors, data analysts) | 11, 12 |
|  | 24b | If blinded, how blinding will be achieved and description of the similarity of interventions | n/a |
|  | 24c | If blinded, circumstances under which unblinding is permissible, and procedure for revealing a participant’s allocated intervention during the trial | n/a |
| **Methods: Data collection, management, and analysis** | | |  |
| Data collection methods | 25a | Plans for assessment and collection of trial data, including any related processes to promote data quality (e.g., duplicate measurements, training of assessors) and a description of trial instruments (e.g., questionnaires, laboratory tests) along with their reliability and validity, if known. Reference to where data collection forms can be accessed, if not in the protocol | 13-15, 20 |
|  | 25b | Plans to promote participant retention and complete follow-up, including list of any outcome data to be collected for participants who discontinue or deviate from intervention protocols | 13-15 |
| Data management | 26 | Plans for data entry, coding, security, and storage, including any related processes to promote data quality (e.g., double data entry; range checks for data values). Reference to where details of data management procedures can be accessed, if not in the protocol | 19, 20 |
| Statistical methods | 27a | Statistical methods used to compare groups for primary and secondary outcomes, including harms | 16, 17 |
|  | 27b | Definition of who will be included in each analysis (e.g., all randomized participants), and in which group | 16-18 |
|  | 27c | How missing data will be handled in the analysis | 17 |
|  | 27d | Methods for any additional analyses (e.g., subgroup and sensitivity analyses) | 15-17 |
| **Methods: Monitoring** | | |  |
| Data monitoring committee | 28a | Composition of data monitoring committee (DMC); summary of its role and reporting structure; statement of whether it is independent from the sponsor and funder; conflicts of interest and reference to where further details about its charter can be found, if not in the protocol. Alternatively, an explanation of why a DMC is not needed | 16, 20 |
|  | 28b | Explanation of any interim analyses and stopping guidelines, including who will have access to these interim results and make the final decision to terminate the trial | 16, 17 |
| Trial monitoring | 29 | Frequency and procedures for monitoring trial conduct. If there is no monitoring, give explanation | 11, 16, 19 |
| **Ethics** | | |  |
| Research ethics approval | 30 | Plans for seeking research ethics committee/institutional review board approval | 18 |
| Protocol amendments | 31 | Plans for communicating important protocol modifications to relevant parties | 19 |
| Consent or assent | 32a | Who will obtain informed consent or assent from potential trial participants or authorized proxies, and how | 9, 19 |
|  | 32b | Additional consent provisions for collection and use of participant data and biological specimens in ancillary studies, if applicable | n/a |
| Confidentiality | 33 | How personal information about potential and enrolled participants will be collected, shared, and maintained in order to protect confidentiality before, during, and after the trial | 19, 20 |
| Ancillary and post-trial care | 34 | Provisions, if any, for ancillary and post-trial care, and for compensation to those who suffer harm from trial participation | 15, 16 |

Citation: Chan A-W, Boutron I, Hopewell S, Moher D, Schulz KF, et al. SPIRIT 2025 statement: updated guideline for protocols of randomised trials. BMJ 2025;389:e081477. <https://dx.doi.org/10.1136/bmj-2024-081477>

**Supplementary 2. PIVO Pro Clinical Skill Assessment Checklist**

| **Performance criteria** | **Assessment (** **as appropriate)** | | | | | |
| --- | --- | --- | --- | --- | --- | --- |
|  | **Date** | | **Date** | | **Date** | |
|  | **Assessor** | | **Assessor** | | **Assessor** | |
|  | **Achieved** | **Not achieved** | **Achieved** | **Not achieved** | **Achieved** | **Not achieved** |
| 1. **Identifies indications and contraindications for blood collection with *PIVO Pro device.*** |  |  |  |  |  |  |
| 2. **Performs hand hygiene.** |  |  |  |  |  |  |
| 3. **Completes peripheral intravenous cannulation insertion as per local procedures.** |  |  |  |  |  |  |
| 4. **Ensures *ExT™ Stabilized Extension Set with NearPort™ IV Access* is primed with saline and secured to PIVC. Connects Needle-free connector and pinches clamp at extension tube.**   **This is required for compatibility with the PIVO Pro device.** |  |  |  |  |  |  |
| 5. **Performs hand hygiene.** |  |  |  |  |  |  |
| 6. **Gathers and prepares necessary equipment for blood collection using PIVO Pro device:**    - PIVO Pro Device    - Saf-T Holder Blood Culture Device – attaches to PIVO Pro    - Alcohol pads    - Tourniquet    - Blood culture bottles    - Saline flush syringe |  |  |  |  |  |  |
| 7. **Disinfects the *NearPort IV Access* with alcohol for 10 seconds and allows it to dry.** |  |  |  |  |  |  |
| 8. **Applies the tourniquet.** |  |  |  |  |  |  |
| 9. **Attaches the PIVO Pro and rotates it so the inner flow tube is visible.** |  |  |  |  |  |  |
| 10. **Slowly pushes the coloured slider forward to advance the inner flow tube through the catheter.**   **Applies light pressure to the top of the ExT stabilisation platform to minimise kinking.** |  |  |  |  |  |  |
| 11. **Attaches the blood collection tubes to the transfer device. Stabilises the slider throughout blood collection.** |  |  |  |  |  |  |
| 12. **Ensures the final blood collection bottle or tube is disconnected from the transfer device.** |  |  |  |  |  |  |
| 13. **Releases the tourniquet.** |  |  |  |  |  |  |
| 14. **Pulls the slider back to withdraw inner tube; then pinches the clip and removes the PIVO Pro device.** |  |  |  |  |  |  |
| 15. **Performs hand hygiene.** |  |  |  |  |  |  |
| 16. **Disinfects the blue MaxZero NFC with alcohol and allow it to dry.** |  |  |  |  |  |  |
| 17. **Unclamps the ExT extension tube, attaches saline flush syringe and pulsatile flushes.** |  |  |  |  |  |  |
| 18. **Removes the saline flush and clamps the extension.** |  |  |  |  |  |  |
| 19. **Performs hand hygiene** |  |  |  |  |  |  |

Connector: *BD ExT™ Stabilized Extension Set with NearPort™*

Blood transfer device: *Saf-T Holder™, ICU Medical*

**Supplementary 3. Verbal consent script**

Hi, my name is [*insert name*], I am a [*research nurse/insert title*] at this hospital working on a research project called ‘The PIVO Trial’.

It is a randomised controlled trial looking at how we take blood cultures – which are blood samples taken to look for infection. Your doctors have ordered blood cultures for you [*or the patient*]. These are usually taken by drawing blood from a peripheral intravenous catheter, or by using a needle inserted through the skin. Taking blood cultures usually involves taking the samples *twice* from two different locations, such as one from a catheter and one with a needle through the skin, to make sure the results are accurate. The PIVO Trial is testing a device (PIVO Pro) which attaches to a peripheral intravenous catheter to take blood cultures, to see if it improves the quality of the sample so we get more accurate results. We will also look at whether taking a blood culture met clinical guidelines and resulted in the best use of antibiotics. All other care is being performed as usual in the hospital.

If you are happy to be in the trial, we will randomly select how your blood cultures are taken. They will be taken either via a newly inserted peripheral intravenous catheter and the PIVO Pro for one sample and the usual hospital approach for the second, or following the usual hospital approach for all blood culture samples you have taken.

The PIVO Pro is used in hospitals overseas as part of standard care, has demonstrated safety and improved care quality, and is approved for use in the USA. The Australian Therapeutic Goods Administration has also approved its use in this trial. Adverse events are not anticipated, and any risk is minimal. There are no direct benefits to you. If you are happy to participate in the trial, we will collect information related to the trial from you and your medical records, but your privacy and confidentiality will be maintained, and your personal details will not be identifiable outside of the hospital. There is no further follow up [*if in substudy 1, instead state “We will come and see you while you are still in hospital to see if you are happy for us to continue to monitor the area where your blood was taken from or your peripheral intravenous catheter”*].

Do you have any questions about the study? [*Answer any questions*].

Do you consent to participate [*or consent to the patient participating*] in this trial? [*If yes, proceed to randomisation; if no, do not proceed*].
